# Expression of Envelope Protein Encoded by Endogenous Retrovirus K102 in Rheumatoid Arthritis Neutrophils

**DOI:** 10.3390/microorganisms11051310

**Published:** 2023-05-17

**Authors:** Amanda Laine, Xiaoxing Wang, Kathryn Ni, Sarah E. B. Smith, Rayan Najjar, Leanne S. Whitmore, Michael Yacoub, Alison Bays, Michael Gale, Tomas Mustelin

**Affiliations:** 1 Division of Rheumatology, Department of Medicine, University of Washington, Seattle, WA 98195, USA; 2 Center for Innate Immunity and Infectious Disease, Department of Immunology, University of Washington, Seattle, WA 98195, USA

**Keywords:** endogenous retrovirus, HERV-K, envelope, autoantibodies, neutrophils, granulocytes

## Abstract

Many patients suffering from autoimmune diseases have autoantibodies against proteins encoded by genomic retroelements, suggesting that normal epigenetic silencing is insufficient to prevent the production of the encoded proteins for which immune tolerance appears to be limited. One such protein is the transmembrane envelope (Env) protein encoded by human endogenous retrovirus K (HERV-K). We reported recently that patients with rheumatoid arthritis (RA) have IgG autoantibodies that recognize Env. Here, we use RNA sequencing of RA neutrophils to analyze HERV-K expression and find that only two loci with an intact open-reading frame for Env, HERV-K102, and K108 are expressed, but only the former is increased in RA. In contrast, other immune cells express more K108 than K102. Patient autoantibodies recognized endogenously expressed Env in breast cancer cells and in RA neutrophils but not healthy controls. A monoclonal anti-Env antibody also detected Env on the surface of RA neutrophils but very little on the surface of other immune cells. We conclude that HERV-K102 is the locus that produces Env detectable on the surface of neutrophils in RA. The low levels of HERV-K108 transcripts may contribute only marginally to cell surface Env on neutrophils or other immune cells in some patients.

## 1. Introduction

Rheumatoid arthritis (RA) is a severe systemic autoimmune disorder affecting ~1% of the population worldwide [1]. The molecular mechanisms that underpin the pathogenesis of RA remain incompletely understood, but it appears that the modification of proteins via arginine deimination (=citrullination) plays an important role by providing neo-epitopes that stoke an escalating immune response against this modified self [2,3]. The association of RA with polymorphisms in the genes that encode two of the citrullinating enzymes, PAD2 [4] and PAD4 [4,5,6,7], further supports the pathogenic role of protein citrullination. Most patients with RA have IgG autoantibodies that recognize citrullinated epitopes, termed anti-citrullinated protein antibodies (ACPA), which are unique to RA [2] and used for its diagnosis [8]. Patients who have ACPA and rheumatoid factor are termed seropositive and usually have classical RA. In contrast, seronegative patients are a more heterogenous group of patients with unspecified inflammatory arthritis that may not be rheumatoid in nature [9].

Another set of autoantibodies reported in RA are those that recognize retroviral group antigen (Gag) and envelope (Env) proteins [10,11,12,13]. These antibodies are present in a subset of patients, but their detection has not been standardized, and the exact antigens they react with remain unclear. Published studies that used proteins from human immunodeficiency virus [14] or short peptides [11,12] from arbitrarily chosen endogenous retroviral loci likely missed other epitopes, potentially resulting in an under-representation of these autoantibodies. Early papers also attempted to clarify which endogenous retroviral loci are expressed in RA patients by detecting their mRNA in RA leukocytes [15], synovial fluid [16,17], or synovial tissue [18]. These studies found increased transcripts derived from the class II human endogenous retroviruses of group K (HERV-K) but did not have the resolution to distinguish which proviruses they originated from.

HERV-K comprise the most recently incorporated retroviruses in our genome, particularly its youngest subgroup termed Human Mouse Mammary Tumor Virus-Like 2 (HML-2), several members of which are human-specific, full-length, and still capable of producing retroviral proteins [19] and even intact virions [20]. That these full-length HML-2 proviruses only recently lost their virulence was best demonstrated by the capacity of a synthetic consensus sequence to produce fully infectious virions that, upon host cell entry, reverse-transcribed their RNA genome and incorporated the resulting DNA into the host cell genome to create new genomic proviruses very similar to the existing HML-2 loci [21]. In contrast, essentially all other endogenized retroviruses in our genome have been incapacitated over evolutionary time by extensive truncations, deletions, insertions, and point-mutations that introduced amino acid changes and stop codons [22], resulting in short open-reading frames (ORFs) that, at best, could be translated into short peptides devoid of their original retroviral functions. Only a handful of HML-2 proviruses are full-length and have intact ORFs for some of the retroviral *gag*, *pro*, *pol*, and *env* encoded proteins. In addition, even recently inserted endogenous retroviruses are silenced by DNA methylation and other epigenetic mechanisms [23] in healthy individuals but can be transcriptionally activated under certain circumstances, e.g., during early embryonic development [24], in cancer [25,26], and in HIV-infected individuals [18,27,28,29,30,31,32,33,34]. Transcripts from many endogenous retrovirus loci are present at elevated levels in patients with systemic lupus erythematosus [35,36], and this correlates with reduced expression of several epigenetic modifiers involved in repressing gene expression.

The presence of anti-HERV-K Env autoantibodies in RA patients suggests that the corresponding protein(s) are, or have been, expressed in this disease. We have determined exactly which HERV-K/HML-2 loci are expressed at increased levels in RA using next-generation RNA sequencing (RNAseq), a technology that yields 101 bp stranded and end-paired sequence reads of essentially all transcripts in a sample. This permits a precise alignment to genomic loci even if they are highly homologous, as is the case within the HERV-K/HML-2 family. Next, we show that RA patient anti-Env antibodies indeed recognize intact, glycosylated, processed Env present on the surface of RA neutrophils.

## 2. Materials and Methods

### 2.1. Human Subjects

Freshly drawn blood from de-identified RA patients (*n* = 30) and healthy controls (*n* = 20) and serum samples from RA patients and healthy controls were obtained from the University of Washington Rheumatology Biorepository with approval of the Institutional Review Board (STUDY00006196). Informed written consent was obtained from all participants according to the Declaration of Helsinki. The demographics and characteristics of the human subjects used in RNA sequencing are shown in Table 1.

### 2.2. Isolation of Polymorphonuclear and Mononuclear Blood Leukocytes

Polymorphonuclear (PMN) and peripheral blood mononuclear cells (PBMC) were isolated from freshly drawn venous blood via gradient centrifugation on PolymorphPrep according to the manufacturer’s instructions. Cells were washed and suspended in Dulbecco’s phosphate-buffered saline at 10^7^/mL.

### 2.3. RNA Isolation and RNA-Seq

Extraction of RNA from patient or healthy control leukocytes (lymphocytes and neutrophils) was performed using Trizol Reagent (Invitrogen cat# 15596026, Waltham, MA, USA) and RNeasy Micro Kit (Qiagen cat. no. 74004, Hilden, Germany). The RNA was precipitated with 70% ethanol, passed through an RNeasy MinElute spin column, and treated with DNase I on the column. RNA purity was verified by A260/A280 and by confirming the presence of the 28S and 18S rRNAs. Further quality controls and RNA-Seq were performed by the Northwest Genomics Center (University of Washington, Seattle, WA, USA).

### 2.4. Bioinformatics of Retrotransposons

FASTQ data were checked for quality using FastQC v0.11.9 and then aligned to the reference human genome GRCh38 using STAR v2.7.9a [37]. Post-alignment QC was conducted using the RSeQC v4.0.0 package [38]. Then, HERVK elements were quantified using TElocal v1.1.1, which is the locus-specific version of TEtranscripts [39] and uses RepeatMasker Open-3.0 annotations. Due to sequence similarity, some transposable elements map to multiple locations in the genome. Therefore, we quantified uniquely mapped elements only. Differential expression testing was performed using DESeq2 1.36.0 [40]. Statistical significance was set at an α = 0.05 with *p* values adjusted for multiple comparisons. Analysis of the publicly available RNA-seq data was performed in a similar way using TElocal v1.1.1, but with alignment to the human genome (hg19), and elements were quantified using the NOISeq R v3.12 package [41]. All genomic sequences with mapping reads were inspected closely, assembled with 5′ and 3′ regions, and translated to determine if an intact envelope-coding open reading frame was present. 

### 2.5. Real-Time and Ordinary Polymerase Chain Reaction (PCR)

cDNA was synthesized from RNA with a poly (dT) primer using QuantiTect Reverse Transcription Kit according to the manufacturer’s instructions. Env and GAPDH PCR was then carried out using a high-fidelity DNA polymerase with K102 Env or K108 Env specific primers K102 forward 5′ AGAAAAGGGCCTCCACGGAGATG, K102 reverse 5′ ATCCTGGTGCTCTCCCTAGG, K108 forward 5′ GTATGCTGCTTGCAGCCTTGATGAT, K108 reverse 5′ GTGACATCCCGCTTACCATG and GAPDH primers forward 5′ CAACGGATTTGGTCGTATT and reverse 5′ GATGGCAACAATATCCACTT, and the Qiagen QuantiTect SYBR Green PCR kit (Qiagen cat# 204143) and ran on an Applied Biosystems StepOne Plus Real-Time PCR Thermal Cycler. The qPCR incorporated ROX dye as a passive reference to normalize for minor variations in fluorescent intensity between reactions, and GAPDH was used as the housekeeping gene.

### 2.6. Affinity-Purification of Patient Antibodies against Env

Due to the poor solubility of our bacterially produced Env-SU protein [13] (except in the presence of agents incompatible with amine-coupling: 6 M Urea, 50 mM Tris, 10 mM DTT, 160 mM L-Arginine, 300 mM NaCl, 10% glycerol, 400 mM Imidazole, pH 8.2), we developed a modified protocol for affinity-purification of patient antibodies binding to it. Briefly, 16.7 µg of Env-SU protein diluted into 1/60 with 0.1 M carbonate coating buffer (pH 9.6) was immobilized on 6-well culture dishes overnight, followed by several washes in phosphate-buffered saline and blocking in 1% bovine serum albumin in phosphate-buffered saline for 2 h. The plates were then incubated with 5 mL RA patient serum per well, washed extensively, and the bound antibodies were eluted at pH 1 and immediately neutralized. A sample of the eluted antibodies was analyzed via SDS gel electrophoresis and Coomassie staining, and the rest were used for immunoblots and directly conjugated with APC-Cyanine7 or AlexaFluor 647 for flow cytometry.

### 2.7. Breast Cancer Cells and Hormone Treatment to Induce HERV-K Expression

The breast cancer cell line T47D was maintained in logarithmic growth in RPMI with 10% fetal calf serum and supplemented with glutamine and antibiotics. To stimulate HERV-K expression [42], the cells were treated overnight with 100 nM progesterone, 10 nM estradiol, or both. The cells were lysed in 20 mM Tris/pH 7.4, 150 mM NaCl, 1% Triton X-100, 5 mM EDTA, and protease inhibitors, clarified by centrifugation and the supernatant mixed with SDS sample buffer and heated to 95 °C for 5 min.

### 2.8. Enrichment in Env from RA Leukocytes

Since functional HERV-K Env has a high affinity for heparan sulfate-containing surface proteins [21], we used heparin-agarose beads to enrich for Env from leukocytes. Briefly, 15 × 10^6^ neutrophils or lymphocytes were lysed in 1 mL ice-cold 20 mM Tris/HCl, pH 7.5, 150 mM NaCl, 5 mM EDTA, 1% NP-40, protease inhibitors (10 µg/mL aprotinin, leupeptin, STI, and PMSF) and centrifugated at 1400 rpm at 0–4 °C for 20 min. A total of 50 µL of 50% heparin-agarose slurry was added to the resulting supernatant, mixed, and incubated on ice for 1 h. This was then pelleted by centrifugation, washed in a lysis buffer 3 times, resuspended in 100 µL of SDS sample buffer, and heated to 95 °C for 2 min.

### 2.9. Transient Expression and Immunofluorescence Staining

293T cells in 4-well Nunc Lab-Tek II CC^2^ chamber slides (ThermoFishe, Waltham, MA, USA) were transfected with 5 µg of Env plasmid (from Dr. Thierry Heidmann) using Lipofectamine 3000 reagents. Then, 48 h post-transfection, cells were fixed with 4% paraformaldehyde and permeabilized in 0.1% saponin. Next, cells were stained with either polyclonal ERVK7 antibody (Thermo Fisher) or monoclonal 22G9 and viewed under an AMG EVOS FL microscope. 

### 2.10. Gel Electrophoresis and Immunoblotting

PMN or PBMC were lysed by mixing 10^7^ cells in 500 µL lysis buffer with an equal volume of twice-concentrated SDS sample buffer, heated at 95 °C, and clarified by centrifugation. A total of 35–45 µL (0.35–0.45 × 10^6^ cell equivalents) samples were resolved by SDS gel electrophoresis and transferred to polyvinylidene fluoride membranes, which were blocked in Superblock in Tris-buffered saline. Filters were incubated with patient-derived anti-Env (1:200), anti-Env monoclonal antibody 22G9 hybridoma supernatant (1:5), patient-derived ACPA monoclonal antibody (1:100) in blocking buffer overnight, washed extensively in Tris-buffered saline with 0.1% Tween-20, and developed with horseradish peroxidase-conjugated anti-human or anti-mouse IgG or IgM and enhanced chemiluminescence detection.

### 2.11. Generation of a Monoclonal Antibody against Env-SU

An IgM mouse monoclonal antibody (22G9) was generated against the 42 kDa SU portion of the Env protein of HERV-K Xq21.33 by Ameritek Inc. (Everett, WA, USA). The resulting hybridoma was adapted to a serum-free medium, and the mAb was purified via ion exchange chromatography (Olympic Protein Technologies LLC, Seattle, WA, USA). Its identity and sequence were determined by mass spectrometry.

### 2.12. Flow Cytometry

Cells were washed twice in phosphate-buffered saline with 1% bovine serum albumin and 1:200 of an unlabeled blocking anti-CD32a antibody, and an excess of unlabeled mouse IgG, and then stained with a mixture of antibodies against surface antigens: anti-CD66b (PE/Cy7-labeled, Biolegend #305115, San Diego, CA, USA) at 1:200, anti-CD16 (PerCP-labeled, clone 3G8 Biolegend #302029) at 1:200, anti-CD14 (PE-labeled anti-human CD14 antibody clone 63D3 (Biolegend #367103) at 1:200, anti-CD19 (APC/Cyanine7-conjugated, clone HIB19, Biolegend #302218) at 1:200, and anti-CD15 (PerCP-conjugated, clone W63D, Biolegend #323018) at 1:200 in phosphate-buffered saline with 1% bovine serum albumin for 30 min at 4 °C in the dark. Affinity-purified patient anti-Env and anti-cit-Env antibodies, or the human mAb L204:01A01, were conjugated to varying AlexaFluor labels according to AlexaFluor Microscale Protein Labeling Kits (Invitrogen A30006, A30009) and added to the cells for 30 min at 4 °C in the dark, washed twice with phosphate-buffered saline, 1% bovine serum albumin, and resuspended in 200 µL of this buffer for analysis on a CytoFLEX Flow Cytometer (Beckman Coulter, Brea, CA, USA). These data were analyzed using BD Bioscience’s FlowJo v10.1 software package.

### 2.13. Statistical Analysis

The statistical significance of the non-parametric data set from patient samples was calculated using the Mann–Whitney U-test. A *p*-value < 0.05 was used as the cut-off for statistical significance; values <0.05 are denoted with *, <0.01 with **, and <0.005 with ***. GraphPad Prism v9 and IBM SPSS software programs were used for all statistical analyses.

## 3. Results

### 3.1. Transcripts from HERV-K Loci That Can Produce Env in RA

**Figure 1:**
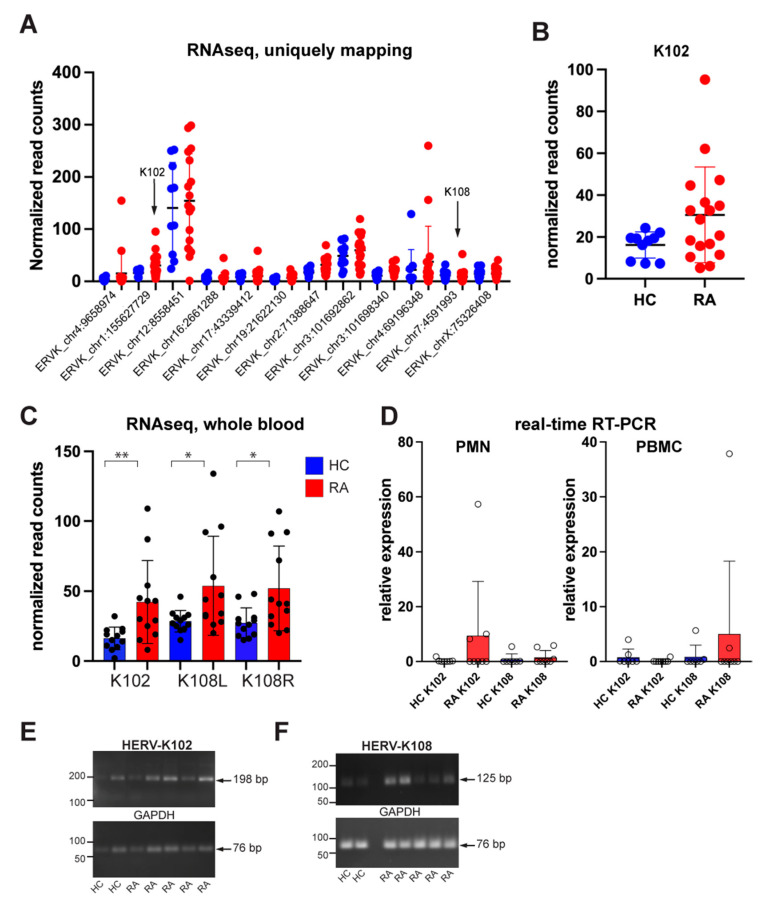
Endogenously expressed HERV-K loci in RA neutrophils. (**A**) All HERV-K transcripts present in neutrophils from RA patients (*n* = 17) and healthy controls (*n* = 10). (**B**) PCR amplification of HERV-K102 and K108 transcripts. (**C**) Independent validation by the GSE90081 RNA-Seq data set of the increased HERV-K102 and K108 transcripts in whole blood from RA patients (red) compared to healthy donors (HC; blue). No other HERV-K locus with an intact open-reading frame for Env was present in this data set. (**D**) Expression of transcriptional regulators of HERV-K, as indicated. (**E**) PCR to confirm K102 amplicon size, (**F**) PCR to confirm K108 amplicon size. * *p* < 0.05, ** *p* < 0.01.

To determine which endogenous HERV-K loci are responsible for the Env protein detectable on the surface of RA patient neutrophils, we isolated RNA from neutrophils from 18 RA patients and 10 healthy controls, followed by RNA-Seq and analysis for transcripts mapping to genomic loci annotated as HERV-K (or its synonyms). Reads uniquely mapped to 177 genomic sequences identified by RepeatMasker were detected in the 28 samples (Supplementary Table S1). Nearly all of these transcriptionally active loci are truncated and extensively mutated, and many contain disrupting *Alu* or other insertions. We first restricted our analysis to those longer than 1600 bp (Table 2, Figure 1A). A closer examination of these loci revealed that only reads uniquely mapping to two full-length loci with intact *env* open-reading frames were present: HERV-K102 (chr1:155,627,729–155,634,877) and K108 (chr7:4,591,993–4,599,432) [43,44]. Importantly, these two transcripts are the only ones with the capacity to be translated into Env proteins with a leader sequence, an extracellular region, a transmembrane helix, and an intracellular tail. Hence, it can be proteolytically processed, glycosylated, and transported to the cell surface. Other loci may only, at best, yield short Env polypeptides predicted to remain intracellular. 

Reads mapping to chr1:155,627,729–155,629,344 correspond to the transcript for K102 *env* and were 1.9-fold higher in RA neutrophils compared to healthy controls (Figure 1B). Reads mapping to the adjacent chr1:155,629,344–155,634,877 correspond to the *gag-pol* region of K102, which contains an in-frame stop codon near the 5′ end of *gag* precluding the translation into full-length Gag, and, hence, no translation of Pol or Pro proteins. Reads from chr7:4,591,993–4,599,432 represent full-length K108, which also has a stop codon in *gag* but could be spliced into an *env*-encoding transcript capable of translation into an intact full-length Env. However, K108 transcripts were low and not increased in RA. The mature Env produced from K102 and K108 are nearly identical (99% identity).

### 3.2. Validation of HERV-K Transcripts in a Public RA Data Set

As an independent verification of the increased HERV-K transcription in RA, we used the publicly available GSE90081 RNA-Seq data set generated from the whole blood of RA patients (*n* = 12) and healthy controls (*n* = 12) [45]. This data set contained increased transcripts from 20 HERV-K loci, as well as decreased transcripts from 13 loci, and unchanged from 5 loci in RA patients compared to healthy volunteers. These data showed that HERV-K102 and K108, but no other transcripts with an intact open-reading frame for Env, were expressed at increased levels in RA compared to healthy controls in a statistically significant manner (Figure 1C). Notably, these data were from whole blood, while ours were from neutrophils, which likely explains the higher levels of K108 transcripts (see below).

### 3.3. Quantitation of K102 and K108 Transcripts in RA Neutrophils and PBMC

Because members of the HERV-K family have varying degrees of sequence similarities, which are particularly high between the younger and more intact members (such as K102 and K108), short-read RNAseq cannot give an accurate estimate of quantity. The results shown in Figure 1A,B and Table 1 refer to ‘uniquely mapping’ reads, i.e., those that do not map to more than one locus. Hence, the subtraction of all reads that map to more than one locus results in an underestimate of reads. For this reason, we turned to quantitative real-time PCR with CYBR Green for more accurate quantitation of HERV-K102 and K108. We used the HERV-K102 primers of Tokuyama et al. [35] and designed primers specific to HERV-K108 that would not amplify transcripts from any of the other HERV-K loci expressed in RA patients. The real-time PCR revealed that K102 was significantly elevated in 3 of 8 RA neutrophil samples but only marginally elevated in 2 of 8 patients and 1 of 7 healthy controls. In contrast, K102 was absent in PBMC, while K108 was present (Figure 1D). Visualizing the PCR products by agarose gel electrophoresis confirmed that the expected 198 bp and 125 bp amplicon were produced (Figure 1E,F). We conclude that neutrophils from RA patients express K102, while K108 is very low in neutrophils but better expressed in other immune lineages present in the PBMC preparation.

### 3.4. Anti-Env Autoantibodies from RA Patients Recognize Cell Surface-Exposed Env

**Figure 2:**
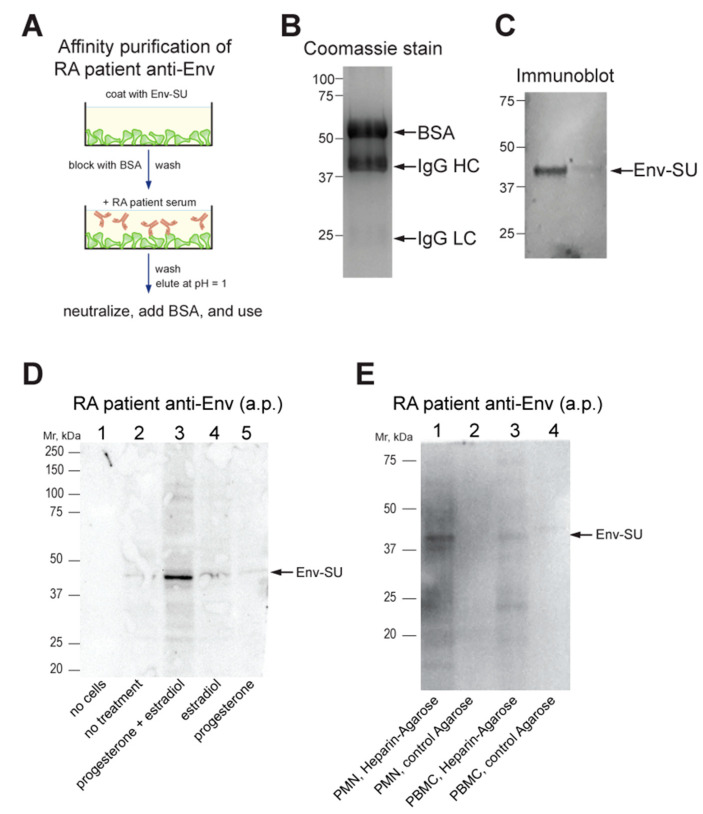
Detection of Env by patient antibodies. (**A**) Schematic illustration of our method of affinity-purification of RA patient anti-Env. (**B**) Coomassie Blue stain of the purified antibodies (with BSA). (**C**) Immunoblot of recombinant Env-SU using the affinity-purified antibodies. (**D**) Immunoblot using the affinity-purified antibodies of T47D breast cancer cells treated without or with hormones as indicated. (**E**) Immunoblot using the affinity-purified antibodies of material enriched by heparin-agarose or control agarose beads from the lysates of RA neutrophils (PMN) or PBMC, as indicated. a.p., affinity-purified; BSA, bovine serum albumin.

We recently reported that RA serum contains IgG autoantibodies that recognize a bacterially produced Env-SU protein [13]. To test if these autoantibodies also recognize endogenously expressed Env, we enriched them via affinity purification using a modified procedure (Figure 2A). Because our recombinant Env-SU protein is not soluble in buffers required for covalent coupling to beads, we instead immobilized it on plastic cell-culture dishes (as in an ELISA), blocked remaining non-specific protein binding with bovine serum albumin (BSA), and washed extensively. After incubation with patient plasma, the plates were extensively washed, and the bound antibodies were eluted with low pH and immediately neutralized. The resulting enriched IgG was visualized by Coomassie Blue staining (Figure 2B), and its ability to recognize Env was validated by immunoblotting (Figure 2C).

To test whether the patient antibodies can recognize endogenously expressed Env, we used the T47D breast cancer cell line, which is known to upregulate HERV-K102 but not K108 [46,47], after treatment with female steroid hormones [42]. Indeed, immunoblots with affinity-purified patient anti-Env of progesterone plus estradiol-treated T47D consistently showed a band at ~43 kDa (Figure 2D), which may represent a subunit of the furin-processed mature Env [48]. There was also a faint band at ~80 kDa in some experiments, which is the size of full-length Env prior to its proteolytic processing.

### 3.5. Endogenously Expressed Env in RA Neutrophils

Because immunoblots of immune cell lysates with the affinity-purified anti-Env gave more non-specific bands (notably Ig heavy chains), we took advantage of the reported affinity of Env for heparan sulfate-containing glycoproteins [49] and enriched immune cell lysates for endogenous Env using heparin-agarose. The eluted material was recognized by patient-derived affinity-purified anti-Env antibodies (Figure 2E).

### 3.6. Generation and Validation of Antibodies Specific for Env for Flow Cytometry

**Figure 3:**
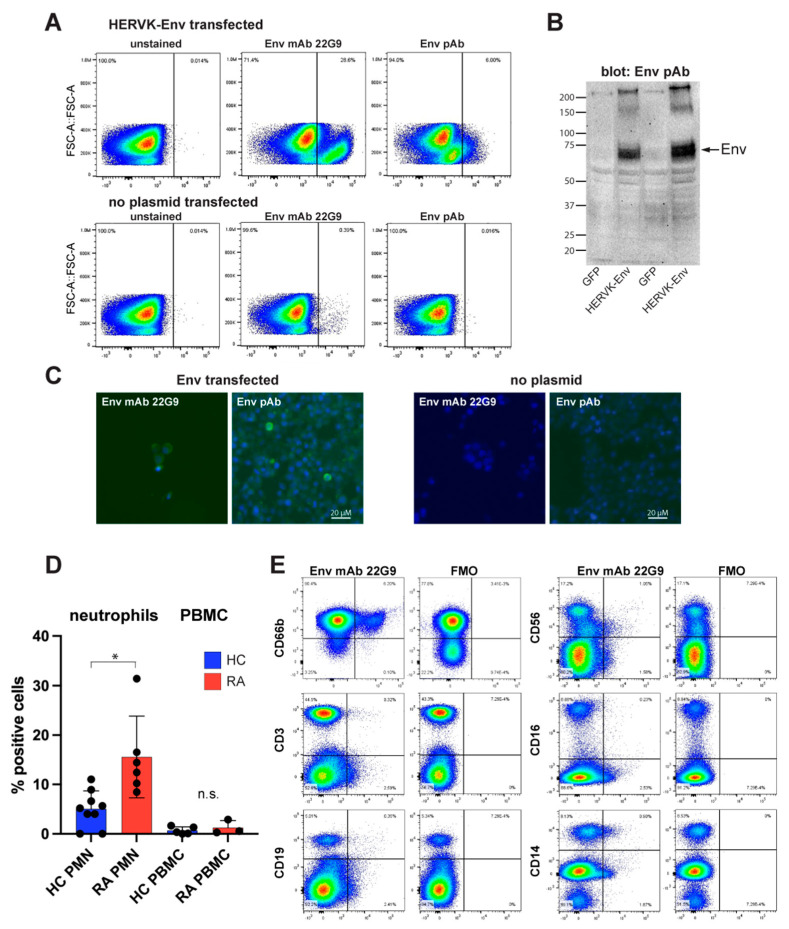
Detection of endogenously expressed Env on immune cells. (**A**) Flow cytometry of Env-transfected (upper panels) or transfected without plasmid (lower panels) stained without antibody (left), fluorophore-conjugated anti-Env mAb 22G9 (middle), or fluorophore-conjugated polyclonal anti-Env (termed anti-ERVK7). (**B**) Control immunoblot of the same transfectants with the polyclonal anti-Env. (**C**) Immunofluorescence microscopy of the same transfected cells (left two panels) using the 22G9 mAb or the polyclonal antibody, as indicated. Right two panels are 293T cells transfected without Env plasmid. (**D**) Summary of flow cytometry with 9 different RA donors (red) and healthy controls (blue) examining neutrophils (PMN) or PBMC. (**E**) A representative set of stains with 22G9 plus lineage markers for neutrophils (CD66b), T lymphocytes (CD3), B lymphocytes (CD19), natural killer cells (CD56), activated monocytes and natural killer cells (CD16), and all monocytes (CD14). FMO, full minus one (i.e., all antibodies except 22G9). * *p* < 0.05.

When directly conjugated with a fluorophore, the affinity-purified patient anti-Env autoantibodies also worked in flow cytometry, but we could not definitively exclude the possibility that these antibodies might bind to other surface proteins. To better demonstrate that the protein recognized by these patient autoantibodies is HERV-K Env, we generated a mouse monoclonal anti-Env IgM (κ light chain) antibody 22G9 via immunization with Env-SU. The hybridoma was adapted to a serum-free medium, and the antibody was purified and verified by partial sequencing by mass spectrometry. Directly fluorophore-labeled 22G9 mAb stained Env-transfected 293T cells brightly without the need for cell permeabilization (Figure 3A), as expected for the transmembrane Env, and with negligible background staining of untransfected cells. A commercial polyclonal antibody against Env (‘ERVK7’), which worked well in immunoblotting, confirmed the expression of Env in the transfected 293T cells (Figure 3B). The 22G9 mAbs also worked in immunofluorescence microscopy (Figure 3C), but not in immunoblotting, perhaps due to being of IgM class. 

### 3.7. Endogenously Expressed Env in Immune Cells from RA Patients

Having validated that the 22G9 mAb anti-Env is specific for Env, we used it to stain non-permeabilized immune cells from RA patients. By isolating both polymorphonuclear and peripheral blood mononuclear cells (PBMC) and analyzing them via a combination of lineage markers and the 22G9 mAb, we observed that neutrophils were the principal lineage expressing Env (Figure 3D,E). An average of 15.8% of RA neutrophils (*n* = 6) were positive compared to 5.0% of neutrophils from healthy donors (*n* = 9; *p* = 0.0026 via Mann–Whitney test) (Figure 3D). In contrast, PBMC from the same donors had very few positive cells, 0.68% in healthy individuals and 1.24% in RA patients (Figure 3D). This small difference was not statistically significant. In the representative flow cytometry shown in Figure 3E, the few Env-positive cells among the PMBC were found to be CD19+ B cells, CD56+ NK cells, and CD14+ monocytes from this RA patient. Their numbers are negligible compared to neutrophils.

## 4. Discussion

For both conceptual and technical reasons, most previous papers on endogenous retroviruses in patients with autoimmune diseases have treated them as families rather than as individual loci. However, even if the exogenous retrovirus that gave rise to the over one hundred HERV-K/HML-2 copies in our genome may have evolved somewhat between the infection events that resulted in the permanent germline insertions that now exist in our genome, these sequences have degenerated in a multitude of ways since then. From the perspective of exploring an anti-HERV-K immune response or possible immunomodulating functions of HERV-K proteins, the most relevant question is whether the expressed loci have intact open-reading frames for proteins that resemble original retroviral proteins. Only some of the youngest loci do, while the vast majority of HERV-K loci do not, well exemplified by the set of transcripts we find in RA patients. These latter transcripts often dominate the repertoire of abnormally represented transcripts, but they are probably of little functional importance unless the transcripts themselves can cause pathology. However, they are produced and processed by the normal host cell machinery and therefore are not very likely to mimic exogenous viral RNAs that can trigger RNA sensors.

The identification of the type 1 HML-2 provirus HERV-K102 as the most likely genomic locus producing the Env protein in the plasma membrane of RA neutrophils is important as it will enable a more precise analysis of factors and mechanisms by which the expression of this provirus is upregulated in this disease. Although we cannot exclude a minor contribution of the K108 locus to Env production, we do not find much evidence that this locus is upregulated in RA neutrophils or that other immune cells that contain a very modest quantity of HERV-K108 transcripts actually express measurable Env on their surface. 

Our finding that HERV-K102 is expressed in RA immune cells is in good agreement with a recent paper from the Iwasaki lab [35], which also found K102 expressed at elevated levels in patients with systemic lupus erythematosus (SLE). We also detected HERV-K102 transcripts in SLE leukocytes [36]. They also reported that transcripts from K106, K110, and K115 were elevated in the patients compared to healthy controls. We did not detect these transcripts in our RA patients but instead detected K108 (albeit very low). Whether this represents a disease-specific difference remains to be determined.

Based on the published literature, we surmise that increased HERV-K expression is not restricted to RA but can accompany other diseases, such as cancer, HIV infection, and other autoimmune conditions, such as systemic lupus erythematosus [35,36]. However, it is already evident that different disease conditions result in the dysregulation of different HERV-K loci. For example, HERV-K108 is reportedly not expressed in breast cancer [47]. Similarly, RA neutrophils express K102, but very little K108, while NK cells and monocytes express K108 but not K102. The reasons for this remain unknown but may relate to the slightly different transcription factor binding sites in the 5′ LTRs of these two proviruses [47].

RA is a disease with a 4:1 ratio of female to male patients. It is, therefore, interesting to note that HERV-K102 is strongly induced by the combination of estradiol and progesterone (Figure 2D), as reported before [42]. Humoral and cellular immunity against HERV-K proteins have also been observed in breast cancer patients [50,51]. Further work will be needed to clarify how similar the (auto)immune responses are in breast cancer compared to RA and to what extent female hormones influence disease progression through upregulating HERV-K expression.

## 5. Conclusions

**Figure 4:**
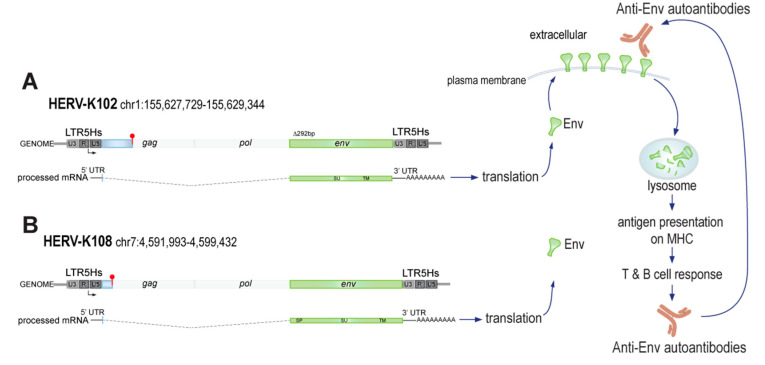
Schematic representation of the HERV-K102 and K108 loci, Env production, and anti-Env generation in RA patients. (**A**) Depicted are the HERV-K102 locus (red club = stop codon), the spliced transcript that encodes for Env, its translation, Env surface transport, and pathway to adaptive immunity leading to anti-Env autoantibodies that can recognize the intact surface-exposed Env. (**B**) The HERV-K108 locus, which has a longer leader sequence in *env*, may possibly contribute to detectable Env in neutrophils and other immune cell lineages.

Our finding that neutrophils from many RA patients express Env on their surface introduces the possibility that this cell lineage is instrumental in the development of anti-Env autoantibodies, which are also present in approximately half of all RA patients [13]. We find that the most likely genomic locus producing the detectable Env protein is HERV-K102 on chromosome 1q22.1 (155,627,729–155,634,877), from which an Env-encoding transcript can be spliced (Figure 4) and translated. It is possible that the HERV-K108 locus contributes to Env production, but this appears to be very minor. Future research will be needed to dissect the transcription of HERV-K102, most likely driven through its 5′ LTR ‘promoter’ region. However, the presence of the Env protein does not entirely match transcript levels, suggesting that translation may also be regulated in RA. Lastly, neutrophils expose the citrullinating enzyme PAD4 on their surface, and we have preliminary data to indicate that Env may be citrullinated, which would explain why patient autoantibodies recognize in vitro citrullinated Env even more strongly than unmodified Env [13]. Future research will explore this possibility.

Our study has an important limitation, namely that the reference genome does not contain all HERV-K proviruses that have been identified but lacks some of the youngest proviruses because they are insertionally polymorphic. They could be present in the genome of some of our human subjects. It is, therefore, possible that we missed HERV-K proviruses that have an intact *env* open reading frame and may contribute, in addition to K102, to the presence of Env on the surface of neutrophils. The Env produced by such proviruses would be near-identical in sequence to Env encoded by K102. 

## Figures and Tables

**Table 1:** Characteristics of the human subjects donating blood for neutrophil RNAseq.

RA Patients (*n* = 17)			
Age (average ± S.D.)	52.1	±14.5	Range 32–73
Sex	4 male	13 female	
Ethnicity/race	9 white, 4 hispanic	1 black, 1 Pacific Is	1 Native Am, 1 n.d. *
Smoking status	2 current	3 former	12 never
CCP/RF #	3 seronegative	14 seroposite	
Erosions	8 not present	9 present	
Clinical Disease Activity Index	10.1	±9.9	Range 0–31
Current medication	4 prednisone, 5 methotrexate	8 anti-TNF, 1 other	1 JAK ihibitor
Healthy controls (*n* = 10)			
Age (average ± S.D.)	35.3	±15.9	Range 21–63
Sex	3 male	7 female	

* n.d., not disclosed’ # CCP = anti-cyclic citrullinated peptide antibody, RF, rheumatoid factor.

**Table 2:** HERV-K reads uniquely mapping to loci > 1600 base pairs in length.

Genomic Locus *	Read Counts in HC	Read Counts in RA	Ratio RA/HC
ERVK_chr1:155,627,729–155,629,344 (K102)	16.2	30.5	1.9
ERVK_chr1:155,629,344–155,634,877 (K102)	168.5	207.9	1.2
ERVK_chr12:8,558,451–8,562,470	140.8	154.5	1.1
ERVK_chr14:69,811,901–69,814,583	25.3	29.4	1.2
ERVK_chr16:2,661,288–2,668,373	4.9	7.1	1.4
ERVK_chr17:43,339,412–43,345,762	8.3	10.1	1.2
ERVK_chr19:21,622,130–21,625,081	2.4	6.8	2.9
ERVK_chr19:58,307,323–58,310,256	271.6	242.2	0.9
ERVK_chr19:58,311,249–58,313,278	151.8	130.8	0.9
ERVK_chr2:71,388,647–71,395,356	17.3	31.9	1.8
ERVK_chr3:101,692,862–101,698,340	48.7	59.5	1.2
ERVK_chr3:101,698,340–101,699,951	10.7	20.6	1.9
ERVK_chr4:153,688,875–153,693,253	24.3	17.4	0.7
ERVK_chr4:69,192,349–69,195,436	13.0	22.4	1.7
ERVK_chr4:69,196,348–69,198,210	22.1	37.5	1.7
ERVK_chr4:9,658,974–9,666,434	3.4	15.1	4.5
ERVK_chr5:1,581,574–1,584,921	23.9	25.4	1.1
ERVK_chr5:71,573,256–71,577,918	12.3	12.3	1.0
ERVK_chr6:29,876,165–29,880,725	45.4	35.5	0.8
ERVK_chr7:4,591,993–4,599,432 (K108)	11.7	8.5	0.7
ERVK_chrX:75,326,408–75,329,359	14.3	15.0	1.0

* as defined by RepeatMasker; some loci may be annotated with two or more adjacent loci.

## Data Availability

The RNAseq data used for this study will be deposited in an NIH-designated data repository with controlled access for general research purposes. All other data in this study will also be made available for research upon request.

## Supplementary Materials

The following supporting information can be downloaded at: https://www.mdpi.com/article/10.3390/microorganisms11051310/s1.
