# Supplementary material for: Expression of Envelope Protein Encoded by Endogenous Retrovirus K102 in Rheumatoid Arthritis Neutrophils
Source: Microorganisms. 2023 May 17;11(5):1310. doi: 10.3390/microorganisms11051310 (PMC10223813; doi:10.3390/microorganisms11051310)
Supplement: Supplementary file 1 [file microorganisms-2375669-supplementary.pdf › microorganisms-2375669-supplementary.pdf]

id	endogenous retrovirus locus	chromos	te.start	te.end	gap from previous	comment	length (nt)	RA1
3	ERVK_chr1:154679406-154679909	chr1	154679406	154679909	65240422		503	3.43127748
4	ERVK_chr1:155627729-155629344	chr1	155627729	155629344	947820	K102 env	1615	11.4375916
5	ERVK_chr1:155629344-155634877	chr1	155629344	155634877	0	K102 gag-pol	5533	191.00778
6	ERVK_chr1:155987603-155988669	chr1	155987603	155988669	352726		1066	3.43127748
7	ERVK_chr1:156179223-156180190	chr1	156179223	156180190	190554		967	21.731424
8	ERVK_chr1:156181461-156182421	chr1	156181461	156182421	1271		960	19.4439057
9	ERVK_chr1:161433900-161434926	chr1	161433900	161434926	5251479		1026	8.00631411
10	ERVK_chr1:161489678-161490610	chr1	161489678	161490610	54752		932	0
11	ERVK_chr1:161536702-161537776	chr1	161536702	161537776	46092		1074	54.9004396
12	ERVK_chr1:161618126-161619201	chr1	161618126	161619201	80350		1075	9.15007327
13	ERVK_chr1:174320521-174321598	chr1	174320521	174321598	12701320		1077	25.1627015
14	ERVK_chr1:192164675-192165673	chr1	192164675	192165673	17843077		998	857.819369
1	ERVK_chr1:77716356-77716832	chr1	77716356	77716832	NA		476	14.8688691
2	ERVK_chr1:89437866-89438984	chr1	89437866	89438984	11721034		1118	0
15	ERVK_chr10:29426711-29427740	chr10	29426711	29427740	NA		1029	5.7187958
16	ERVK_chr10:45488476-45488949	chr10	45488476	45488949	16060736		473	3.43127748
17	ERVK_chr10:45999335-46000000	chr10	45999335	46000000	510386		665	10.2938324
18	ERVK_chr10:46000001-46000405	chr10	46000001	46000405	1		404	10.2938324
19	ERVK_chr10:46094952-46095291	chr10	46094952	46095291	94547		339	40.0315706
20	ERVK_chr10:72881948-72883016	chr10	72881948	72883016	26786657		1068	10.2938324
21	ERVK_chr10:89398090-89398543	chr10	89398090	89398543	16515074		453	26.3064607
22	ERVK_chr10:97416846-97417535	chr10	97416846	97417535	8018303		689	9.15007327
23	ERVK_chr10:97417835-97418118	chr10	97417835	97418118	300		283	2.28751832
28	ERVK_chr11:121568951-121570035	chr11	121568951	121570035	119282059		1084	38.8878114
27	ERVK_chr11:2285884-2286892	chr11	2285884	2286892	1930544		1008	30.8814973
24	ERVK_chr11:324822-325786	chr11	324822	325786	NA		964	72.056827
25	ERVK_chr11:354528-355122	chr11	354528	355122	28742		594	4.57503664
26	ERVK_chr11:355293-355340	chr11	355293	355340	171		47	3.43127748
34	ERVK_chr12:121280127-121281204	chr12	121280127	121281204	26672536		1077	4.57503664
29	ERVK_chr12:6741889-6742530	chr12	6741889	6742530	NA		641	41.1753297
30	ERVK_chr12:8141232-8142314	chr12	8141232	8142314	1398702		1082	66.3380312
31	ERVK_chr12:8537686-8538696	chr12	8537686	8538696	395372		1010	13.7251099
32	ERVK_chr12:8558451-8562470	chr12	8558451	8562470	19755		4019	181.857706
33	ERVK_chr12:94606559-94607591	chr12	94606559	94607591	86044089		1032	69.7693087
36	ERVK_chr13:113450366-113451381	chr13	113450366	113451381	93632544		1015	2.28751832
37	ERVK_chr13:113621367-113622349	chr13	113621367	113622349	169986		982	14.8688691
35	ERVK_chr13:19817339-19817822	chr13	19817339	19817822	NA		483	9.15007327
44	ERVK_chr14:102410264-102410371	chr14	102410264	102410371	31461824		107	25.1627015
38	ERVK_chr14:24201967-24203033	chr14	24201967	24203033	NA		1066	13.7251099
39	ERVK_chr14:30562170-30563147	chr14	30562170	30563147	6359137		977	9.15007327
40	ERVK_chr14:69811901-69814583	chr14	69811901	69814583	39248754		2682	37.7440522
41	ERVK_chr14:69814767-69815504	chr14	69814767	69815504	184		737	14.8688691
42	ERVK_chr14:69815602-69816023	chr14	69815602	69816023	98		421	0
43	ERVK_chr14:70947364-70948440	chr14	70947364	70948440	1131341		1076	29.7377381
45	ERVK_chr15:40688031-40688266	chr15	40688031	40688266	NA		235	14.8688691
46	ERVK_chr15:40689452-40690090	chr15	40689452	40690090	1186		638	59.4754763
47	ERVK_chr15:51612383-51613441	chr15	51612383	51613441	10922293		1058	13.7251099
48	ERVK_chr15:75190420-75190927	chr15	75190420	75190927	23576979		507	1.14375916
49	ERVK_chr16:2661288-2668373	chr16	2661288	2668373	NA		7085	5.7187958
50	ERVK_chr16:3078991-3080484	chr16	3078991	3080484	410618		1493	139.538617
51	ERVK_chr16:3080481-3081056	chr16	3080481	3081056	-3		575	19.4439057
52	ERVK_chr16:3081057-3081509	chr16	3081057	3081509	1		452	41.1753297
53	ERVK_chr16:53384166-53385236	chr16	53384166	53385236	50302657		1070	19.4439057
54	ERVK_chr16:87864600-87865066	chr16	87864600	87865066	34479364		466	4.57503664
55	ERVK_chr16:89752454-89753458	chr16	89752454	89753458	1887388		1004	22.8751832
56	ERVK_chr17:18302964-18303938	chr17	18302964	18303938	NA		974	24.0189423
57	ERVK_chr17:30700041-30701008	chr17	30700041	30701008	12396103		967	16.0126282
58	ERVK_chr17:43339412-43345762	chr17	43339412	43345762	12638404		6350	17.1563874
59	ERVK_chr17:43372204-43373029	chr17	43372204	43373029	26442		825	3.43127748
60	ERVK_chr17:46283614-46284581	chr17	46283614	46284581	2910585		967	0
62	ERVK_chr18:21784426-21785421	chr18	21784426	21785421	12255833		995	8.00631411
61	ERVK_chr18:9528074-9528593	chr18	9528074	9528593	NA		519	4.57503664
66	ERVK_chr19:21622130-21625081	chr19	21622130	21625081	14747736		2951	5.7187958
63	ERVK_chr19:3175888-3176369	chr19	3175888	3176369	NA		481	41.1753297
67	ERVK_chr19:40822624-40823609	chr19	40822624	40823609	19197543		985	9.15007327
68	ERVK_chr19:41799986-41800994	chr19	41799986	41800994	976377		1008	377.440522
69	ERVK_chr19:46768372-46769368	chr19	46768372	46769368	4967378		996	13.7251099
70	ERVK_chr19:51413867-51414365	chr19	51413867	51414365	4644499		498	14.8688691
71	ERVK_chr19:51730738-51731812	chr19	51730738	51731812	316373		1074	13.7251099
72	ERVK_chr19:51960243-51961231	chr19	51960243	51961231	228431		988	10.2938324
73	ERVK_chr19:52577366-52577973	chr19	52577366	52577973	616135		607	9.15007327
74	ERVK_chr19:54893402-54893878	chr19	54893402	54893878	2315429		476	52.6129213
75	ERVK_chr19:57810330-57811158	chr19	57810330	57811158	2916452		828	45.7503664
76	ERVK_chr19:57811462-57811568	chr19	57811462	57811568	304		106	4.57503664
77	ERVK_chr19:58306515-58307228	chr19	58306515	58307228	494947		713	74.3443453
78	ERVK_chr19:58307323-58310256	chr19	58307323	58310256	95		2933	331.690156
79	ERVK_chr19:58311249-58313278	chr19	58311249	58313278	993		2029	101.794565
80	ERVK_chr19:58313591-58314344	chr19	58313591	58314344	313		753	108.65712
81	ERVK_chr19:58584237-58585172	chr19	58584237	58585172	269893		935	50.325403
64	ERVK_chr19:6868975-6869936	chr19	6868975	6869936	3692606		961	20.5876649
65	ERVK_chr19:6873938-6874394	chr19	6873938	6874394	4002		456	21.731424
87	ERVK_chr2:152754873-152755883	chr2	152754873	152755883	55013625		1010	10.2938324
88	ERVK_chr2:186521529-186522372	chr2	186521529	186522372	33765646		843	2.28751832
89	ERVK_chr2:218144566-218145075	chr2	218144566	218145075	31622194		509	12.5813508
90	ERVK_chr2:237786592-237786795	chr2	237786592	237786795	19641517		203	17.1563874
91	ERVK_chr2:237786779-237787243	chr2	237786779	237787243	-16		464	61.7629946
82	ERVK_chr2:48384541-48385019	chr2	48384541	48385019	NA		478	8.00631411
83	ERVK_chr2:71388099-71388644	chr2	71388099	71388644	23003080		545	112.088398
84	ERVK_chr2:71388647-71395356	chr2	71388647	71395356	3		6709	19.4439057
85	ERVK_chr2:71444434-71445593	chr2	71444434	71445593	49078		1159	33.1690156
86	ERVK_chr2:97740823-97741248	chr2	97740823	97741248	26295230		425	12.5813508
92	ERVK_chr20:34424999-34425992	chr20	34424999	34425992	NA		993	5.7187958
93	ERVK_chr20:35262124-35263091	chr20	35262124	35263091	836132		967	21.731424
94	ERVK_chr20:45088417-45088913	chr20	45088417	45088913	9825326		496	57.187958
95	ERVK_chr20:49293330-49294380	chr20	49293330	49294380	4204417		1050	50.325403
96	ERVK_chr22:19319891-19320930	chr22	19319891	19320930	NA		1039	2.28751832
97	ERVK_chr22:42551535-42552521	chr22	42551535	42552521	23230605		986	0
98	ERVK_chr22:45169406-45170372	chr22	45169406	45170372	2616885		966	14.8688691
99	ERVK_chr22:50533868-50534815	chr22	50533868	50534815	5363496		947	110.944638
105	ERVK_chr3:101692862-101698340	chr3	101692862	101698340	54232335		5478	72.056827
106	ERVK_chr3:101698340-101699951	chr3	101698340	101699951	0		1611	12.5813508
107	ERVK_chr3:122443536-122444603	chr3	122443536	122444603	20743585		1067	30.8814973
108	ERVK_chr3:129850601-129851115	chr3	129850601	129851115	7405998		514	22.8751832
109	ERVK_chr3:170077867-170078720	chr3	170077867	170078720	40226752		853	9.15007327
110	ERVK_chr3:170078835-170079290	chr3	170078835	170079290	115		455	1.14375916
111	ERVK_chr3:196683050-196684092	chr3	196683050	196684092	26603760		1042	17.1563874
112	ERVK_chr3:196944080-196945028	chr3	196944080	196945028	259988		948	10.2938324
100	ERVK_chr3:19987775-19988838	chr3	19987775	19988838	NA		1063	245.908219
101	ERVK_chr3:30706193-30706684	chr3	30706193	30706684	10717355		491	16.0126282
102	ERVK_chr3:38135237-38136292	chr3	38135237	38136292	7428553		1055	13.7251099
103	ERVK_chr3:38202544-38203501	chr3	38202544	38203501	66252		957	8.00631411
104	ERVK_chr3:47459416-47460527	chr3	47459416	47460527	9255915		1111	3.43127748
118	ERVK_chr4:102718684-102719555	chr4	102718684	102719555	33519450		871	5.7187958
119	ERVK_chr4:153688875-153693253	chr4	153688875	153693253	50969320		4378	19.4439057
120	ERVK_chr4:168993975-168994479	chr4	168993975	168994479	15300722		504	17.1563874
114	ERVK_chr4:26315207-26316247	chr4	26315207	26316247	16648773		1040	12.5813508
115	**ERVK_chr4:69192349-69195436**	chr4	69192349	69195436	42876102		3087	14.8688691
116	ERVK_chr4:69196348-69198210	chr4	69196348	69198210	912		1862	25.1627015
117	ERVK_chr4:69198211-69199234	chr4	69198211	69199234	1		1023	19.4439057
113	ERVK_chr4:9658974-9666434	chr4	9658974	9666434	NA		7460	4.57503664
128	ERVK_chr5:138410509-138411512	chr5	138410509	138411512	57938787		1003	4.57503664
129	ERVK_chr5:150536986-150537953	chr5	150536986	150537953	12125474		967	100.650806
130	ERVK_chr5:151729381-151730458	chr5	151729381	151730458	1191428		1077	73.2005862
121	ERVK_chr5:1579846-1580839	chr5	1579846	1580839	NA		993	52.6129213
122	ERVK_chr5:1581574-1584921	chr5	1581574	1584921	735		3347	52.6129213
131	ERVK_chr5:179990016-179991019	chr5	179990016	179991019	28259558		1003	85.7819369
123	ERVK_chr5:43024822-43025872	chr5	43024822	43025872	41439901		1050	3.43127748
124	ERVK_chr5:43058135-43059111	chr5	43058135	43059111	32263		976	37.7440522
125	ERVK_chr5:58513412-58514371	chr5	58513412	58514371	15454301		959	0
126	ERVK_chr5:71573256-71577918	chr5	71573256	71577918	13058885		4662	11.4375916
127	ERVK_chr5:80470850-80471722	chr5	80470850	80471722	8892932		872	13.7251099
143	ERVK_chr6:132720806-132721774	chr6	132720806	132721774	48724556		968	2771.32844
144	ERVK_chr6:166915130-166915981	chr6	166915130	166915981	34193356		851	101.794565
145	ERVK_chr6:166950876-166951859	chr6	166950876	166951859	34895		983	122.38223
133	ERVK_chr6:17814837-17815833	chr6	17814837	17815833	14805103		996	66.3380312
134	ERVK_chr6:25981969-25982575	chr6	25981969	25982575	8166136		606	45.7503664
135	ERVK_chr6:26433302-26434360	chr6	26433302	26434360	450727		1058	5.7187958
136	ERVK_chr6:27825846-27826625	chr6	27825846	27826625	1391486		779	30.8814973
137	ERVK_chr6:29876165-29880725	chr6	29876165	29880725	2049540		4560	67.4817904
132	ERVK_chr6:3008666-3009734	chr6	3008666	3009734	NA		1068	6.86255495
138	ERVK_chr6:31263182-31264217	chr6	31263182	31264217	1382457		1035	16.0126282
139	ERVK_chr6:32655747-32656815	chr6	32655747	32656815	1391530		1068	42.3190889
140	ERVK_chr6:32657126-32658083	chr6	32657126	32658083	311		957	35.4565339
141	ERVK_chr6:35559719-35560716	chr6	35559719	35560716	2901636		997	2.28751832
142	ERVK_chr6:83995182-83996250	chr6	83995182	83996250	48434466		1068	75.4881045
150	ERVK_chr7:100217447-100218453	chr7	100217447	100218453	24803577		1006	58.3317171
147	ERVK_chr7:30509034-30509894	chr7	30509034	30509894	25909602		860	5.7187958
146	ERVK_chr7:4591993-4599432	chr7	4591993	4599432	NA	full K108 w env	7439	4.57503664
148	ERVK_chr7:66662972-66663403	chr7	66662972	66663403	36153078		431	2.28751832
149	ERVK_chr7:75412888-75413870	chr7	75412888	75413870	8749485		982	13.7251099
156	ERVK_chr8:100707203-100708209	chr8	100707203	100708209	26665066		1006	259.633329
153	ERVK_chr8:12565980-12567003	chr8	12565980	12567003	5065980		1023	34.3127748
157	ERVK_chr8:129858912-129859911	chr8	129858912	129859911	29150703		999	25.1627015
158	ERVK_chr8:132723826-132724841	chr8	132723826	132724841	2863915		1015	27.4502198
159	ERVK_chr8:132741308-132742314	chr8	132741308	132742314	16467		1006	148.688691
160	ERVK_chr8:143849709-143850662	chr8	143849709	143850662	11107395		953	0
161	ERVK_chr8:144897564-144898083	chr8	144897564	144898083	1046902		519	13.7251099
154	ERVK_chr8:30622180-30623246	chr8	30622180	30623246	18055177		1066	8.00631411
151	ERVK_chr8:684634-685715	chr8	684634	685715	NA		1081	2.28751832
155	ERVK_chr8:74041023-74042137	chr8	74041023	74042137	43417777		1114	11.4375916
152	ERVK_chr8:7498938-7500000	chr8	7498938	7500000	6813223		1062	5.7187958
165	ERVK_chr9:128518888-128519972	chr9	128518888	128519972	32441746		1084	59.4754763
166	ERVK_chr9:136796533-136797430	chr9	136796533	136797430	8276561		897	16.0126282
162	ERVK_chr9:90901445-90902406	chr9	90901445	90902406	NA		961	28.593979
163	ERVK_chr9:90904718-90906293	chr9	90904718	90906293	2312		1575	10.2938324
164	ERVK_chr9:96076138-96077142	chr9	96076138	96077142	5169845		1004	2.28751832
170	ERVK_chrX:149491847-149492285	chrX	149491847	149492285	74162488		438	36.6002931
167	ERVK_chrX:53159114-53159544	chrX	53159114	53159544	NA		430	12.5813508
168	ERVK_chrX:53159659-53160870	chrX	53159659	53160870	115		1211	16.0126282
169	ERVK_chrX:75326408-75329359	chrX	75326408	75329359	22165538		2951	9.15007327
172	ERVK_chrY:21180645-21180948	chrY	21180645	21180948	14430790		303	0
173	ERVK_chrY:21181904-21183246	chrY	21181904	21183246	956		1342	0
171	ERVK_chrY:6748889-6749855	chrY	6748889	6749855	NA		966	9.15007327
